# Thermogenic Effect of Glucose in Hypothyroid Subjects

**DOI:** 10.1155/2014/308017

**Published:** 2014-03-10

**Authors:** Agnieszka Kozacz, Paulina Grunt, Marta Steczkowska, Tomasz Mikulski, Jan Dąbrowski, Monika Górecka, Urszula Sanocka, Andrzej Wojciech Ziemba

**Affiliations:** ^1^Mossakowski Medical Research Center, Polish Academy of Sciences, 5 Pawinskiego Street, 02-106 Warsaw, Poland; ^2^Masovian Hospital Bródno, 8 Kondratowicza Street, 03-242 Warsaw, Poland

## Abstract

The importance of thyroid hormone, catecholamines, and insulin in modification of the thermogenic effect of glucose (TEG) was examined in 34 healthy and 32 hypothyroid subjects. We calculated the energy expenditure at rest and during oral glucose tolerance test. Blood samples for determinations of glucose, plasma insulin, adrenaline (A), and noradrenaline (NA) were collected. It was found that TEG was lower in hypothyroid than in control group (19.68 ± 3.90 versus 55.40 ± 7.32 kJ, resp., *P* < 0.0004). Mean values of glucose and insulin areas under the curve were higher in women with hypothyroidism than in control group (286.79 ± 23.65 versus 188.41 ± 15.84 mmol/L*·*min, *P* < 0.003 and 7563.27 ± 863.65 versus 4987.72 ± 583.88 mU/L*·*min, *P* < 0.03 resp.). Maximal levels of catecholamines after glucose ingestion were higher in hypothyroid patients than in control subjects (Amax—0.69 ± 0.08 versus 0.30 ± 0.07 nmol/L, *P* < 0.0001, and NAmax—6.42 ± 0.86 versus 2.54 ± 0.30 nmol/L, *P* < 0.0002). It can be concluded that in hypothyroidism TEG and glucose tolerance are decreased while the adrenergic response to glucose administration is enhanced. Presumably, these changes are related to decreased insulin sensitivity and responsiveness to catecholamine action.

## 1. Introduction

Postprandial thermogenesis is an important component (*≈*10%) of total daily energy expenditure. Each ingested meal increases heat production and induces the thermogenic effect. Thermogenic effect of glucose administration (TEG) is often used as a model to study postprandial thermogenesis. Insulin and adrenergic stimulation are the primary factors involved in postprandial thermogenesis. TEG decreases in the presence of insulin resistance and/or reduces insulin response to glucose load [1, 2]. Insulin resistance decreases TEG because of lower rate of glucose uptake by the tissues (Insulin-dependent glucose transport) and hence decreases the rate of glucose storage, which is an energy-requiring process [1]. Acheson et al. [3] concluded that “approximately 70% of the thermogenic response to glucose infusion is related to the energy cost of glucose storage while the remaining 30% is mediated via calorigenic action of *β*-adrenergic sympathetic nervous activity.” Blockade of peripheral *β*-adrenergic receptor results in a significant reduction in thermic effect of food (TEF) [3–5], demonstrating the importance of sympathetic nervous system in mediation of TEF (although this has not been observed in all studies [6]). Thyroid hormone plays a key role in regulation of the basal metabolic rate; however, its impact on postprandial thermogenesis is still poorly understood [7–9]. Thyroid hormone is an important determinant of glucose homeostasis. Disorders of carbohydrate metabolism, closely associated with overt forms of thyroid dysfunction, are manifested by impaired glucose tolerance. Both hypothyroidism and hyperthyroidism affect insulin sensitivity [10–14]. Thyroid hormone is essential in maximizing the responsiveness to catecholamines at adrenergic receptor level as well as at several postreceptor steps in the catecholamine signaling pathways (particularly those initiated in the *β*-adrenergic receptors) [15–17]. The purpose of the study is to make an integrative assessment of interrelationships between thyroid hormone, adrenergic system, and insulin in modulation of postprandial thermogenesis as well as carbohydrate metabolism. This will provide answers to questions: (1) is glucose-induced postprandial thermogenesis different in hypothyroid patients than in healthy people? (2) Does adrenergic response to glucose load in hypothyroid patients differ from the response of healthy people?

## 2. Research Methods and Procedures

### 2.1. Study Population

The study population consisted of 32 women (mean age 38.5 ± 2.38 years, mean BMI 28.9 ± 0.91 kg/m^2^) with newly diagnosed and never treated hypothyroidism who were consecutively included in the study. The subjects' hypothyroidism varied in etiology and included, for example, iodine deficiency, infections, and thyroiditis (including postpartum, subacute, silence thyroiditis, and Hashimoto's thyroiditis). The diagnoses were based on (1) medical history identifying among others: rapid weight gain, oedema, fatigue, mood deterioration, constipation, skin dryness, and discoloration around elbows and knees; (2) TSH, fT3, and fT4 determination antibodies against specific thyroid antigens; and (3) thyroid ultrasonography. The control group consisted of 34 healthy women without any thyroid disorders (mean age 38.1 ± 1.85 years, mean BMI 27.7 ± 0.86 kg/m^2^) matched for age and BMI, recruited by the Department of Applied Physiology, Medical Research Center, Polish Academy of Sciences, Warsaw. Subjects were excluded from the study if they had serious metabolic diseases and dysfunctions that could affect the test results (diseases that affect the basic energy expenditure, such as diabetes, serious heart disease, and liver failure). All participants gave their informed consent to the study which was approved by the Local Ethics Committee of the Medical University of Warsaw.

### 2.2. Study Design

The study was conducted in a specially prepared unit at the Department of Applied Physiology, in an air-conditioned room with ambient temperature kept between 22 and 24°C. The women reported to the Department in the morning following an overnight fast. Intravenous cannula was inserted into the antecubital vein and the first blood sample was taken after a 30 min bed rest. Afterwards, the supine subjects were submitted to the 120 min oral glucose tolerance test (OGTT). For this purpose they drank 75 g of glucose dissolved in 200 mL of lukewarm water. Venous blood samples were analyzed for plasma insulin (IRI) and blood glucose (BG) immediately prior to and at 30th, 60th, 90th, and 120th min following glucose ingestion and for plasma catecholamines before glucose ingestion and at 90th, and 120th min. It was demonstrated by Mathias et al. in 1989 that the highest level of plasma catecholamines during OGTT is ascertained in the second hour after glucose load [18]. Measurements of energy expenditure were carried out 20 minutes before administration of glucose and then for 5 minutes every fifteen minutes during the OGTT. Measurements of energy expenditure were performed by using an indirect calorimetry. Oxygen uptake (VO_2_) and carbon dioxide production (VCO_2_) were determined with the Vmax29-SensorMedics (CareFusion, San Diego, California, USA) gas analyzer, with the accuracy of ±0.02% for O_2_ and ±0.02% for CO_2_. Energy expenditure was calculated using mean values of VO_2_ and VCO_2_ recorded for 20 min before glucose ingestion (RMR) or for 5 min every 15 min following glucose load. Resting metabolic rate was expressed in kJ/h/kg. The increase in energy expenditure induced by glucose ingestion, that is, the thermogenic effect of glucose (TEG), was calculated as incremental area under the curve obtained during the 2-hour period following glucose load.

### 2.3. Analytical Procedures

Blood glucose (BG) concentration was measured with glucometer One Touch Profile (Lifescan, Milpitas, California, USA). Plasma insulin (IRI) was assessed by radioimmunoassay with a reagent kit MI-130 (Research and Development Centre of Isotopes POLATOM, Świerk, Poland). Plasma adrenaline (A) and noradrenaline (NA) levels were determined by radioimmunoassay with a reagent kit 2-CAT RIA (DIAsource ImmunoAssays S.A., Louvain-la-Neuve, Belgium). The integrated responses of BG and plasma IRI to the glucose load were expressed as incremental areas under their respective time curves (auc). Insulin sensitivity was estimated in four ways: (1) by fasting insulin level, (2) the homoeostasis model of insulin resistance (HOMA-IR)—{HOMA-IR = fasting plasma insulin (mU/L) × fasting plasma glucose (mmol/L)/22.5}, (3) the quantitative insulin sensitivity check index (QUICKI)—{QUICKI = 1/(log(fasting insulin (*μ*U/mL)) + log(fasting glucose (mg/dL)))}, and (4) the Matsuda index—(Matsuda index = 10 000/SQRT (fasting glucose (mmol/L) × fasting insulin (mU/L) × mean glucose_(0–120)_ (mmol/L) × mean insulin_(0–120)_ (mU/L))).

### 2.4. Statistical Analysis

To assess the normality of variables the Shapiro-Wilk test was used. Statistical analysis included two-way analysis of variance (ANOVA) followed by multiple comparatory tests (Newman-Keuls post hoc test) and Levene and Brown-Forsythe test for homogeneity of variance. To compare values between the control and the test group, the Student's *t*-test, Cochran and Cox test, or Mann-Whitney* U* test were used when appropriate. The Wilcoxon signed-ranktest was used to compare data obtained within a single group. Statistical analyses were made using Statistica 5 (StatSoft). *P* values of 0.05 or less were considered statistically significant. Group data in the text, table, and figures are presented as the mean values ± standard error (±SE).

## 3. Results

Thirty-two female subjects suffering from hypothyroidism were studied and thirty-four healthy women served as a control group. The women were matched as closely as possible. The group anthropometric characteristics were comparable while all the other study parameters presented in Table 1 were significantly different (Table 1).

### 3.1. Resting Metabolic Rate and Changes in Energy Expenditure during a 2-Hour Period after Glucose Ingestion

**Figure 1:**
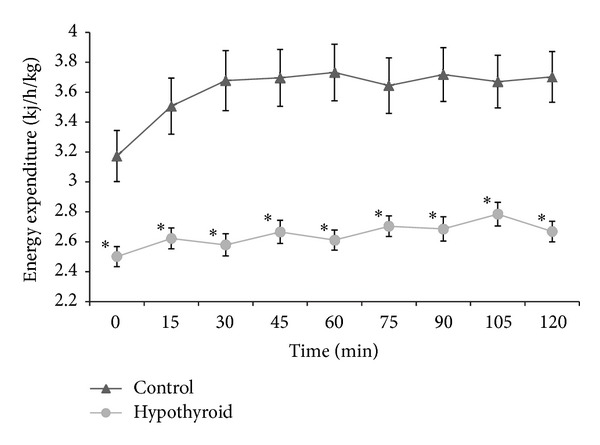
Mean values ± SE of RMR and changes in energy expenditure during 2 h OGTT in the group of hypothyroid (hypothyroid) and healthy women (control). Asterisks denote differences between these groups; *P < 0.0001.

Two-way analysis of variance demonstrated significant hypothyroid or healthy group effects (*P* < 0.0001), the time factor (*P* < 0.0001), and the interaction of these two factors (*P* < 0.0002). Average value of the RMR was lower in hypothyroid patients than in the control group (*P* < 0.0001). After glucose ingestion, energy expenditure (Figure 1) increased in both groups and remained elevated throughout the duration of the test. However, in healthy women, fast significant increase in metabolic rate occurred in the first 15 min (*P* < 0.0001) and maintained significantly elevated until the end of the experiment. By contrast, in the group of patients with hypothyroidism, there was only a slight increase in energy expenditure which was statistically insignificant except for the maximum level appearing at 105th min (*P* < 0.003). The maximum value of energy expenditure in the group of healthy individuals was achieved 1 h after administration of glucose, while in the group with hypothyroidism it occurred only at 105th min of the test and was significantly lower (*P* < 0.0001) than in the control group. The values of energy expenditure during all measurements were significantly lower in women with hypothyroidism (*P* < 0.0001). Consequently, a small increase in energy expenditure of hypothyroid women caused the value of TEG to be more than twice as low in hypothyroid group 19.68 ± 3.90 kJ than in control group 55.40 ± 7.32 kJ (*P* < 0.0004).

### 3.2. Blood Glucose and Plasma Insulin Responses to the Glucose Load

**Figure 2:**
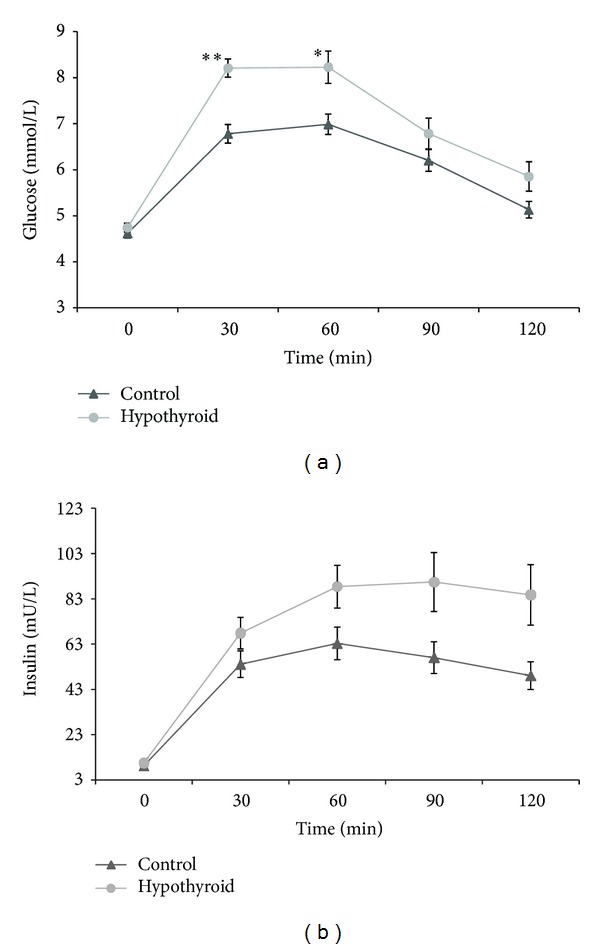
Mean values ± SE of fasting blood glucose and insulin and their postprandial changes in the group of hypothyroid (hypothyroid) and healthy women (control). Asterisks denote differences between these groups; *P < 0.005; **P < 0.0001.

There were no statistically significant differences in fasting blood glucose and fasting insulin between the groups. Oral administration of glucose in both groups caused expected fluctuations in blood glucose and insulin (Figure 2). Blood glucose in both groups rose rapidly and was significantly higher in the first measurement (*P* < 0.0001) than in the fasting state. The maximal level of blood glucose was reached 1 hour after administration of glucose in both groups and it was significantly higher in women with thyroid disorder (*P* < 0.005). Subsequently, the glucose level dropped; however, after 2 h of the test, it was still higher than in the fasting state in both healthy women (*P* < 0.003) and hypothyroid patients (*P* < 0.0001).

Plasma insulin levels during the test tended to be higher in women with hypothyroidism than in the healthy subjects until the end of the test but the differences were not significant. As in the case of glucose, insulin rose rapidly; the level of insulin in the first measurement was statistically different from fasting state (*P* < 0.0001) in both groups. Maximal values of plasma insulin did not differ between the groups; however, they were achieved more rapidly by healthy women (at 60th minute of the test) than by women with hypothyroidism (90th min). In fact, insulin levels even increased in the group with hypothyroidism when in the control group they had already fallen. At the end of the OGTT, insulin levels remained significantly elevated in both groups compared to baseline values (*P* < 0.0001). Calculated areas under the curves of blood glucose and plasma insulin in the OGTT were significantly higher in women with hypothyroidism than in women without thyroid hormone deficiency.

Calculated indicators of insulin resistance were not significantly different between groups (Table 2) and were on the verge of defining insulin or indicated insulin resistance for both groups.

### 3.3. Plasma Catecholamine Responses to the Glucose Load

**Figure 3:**
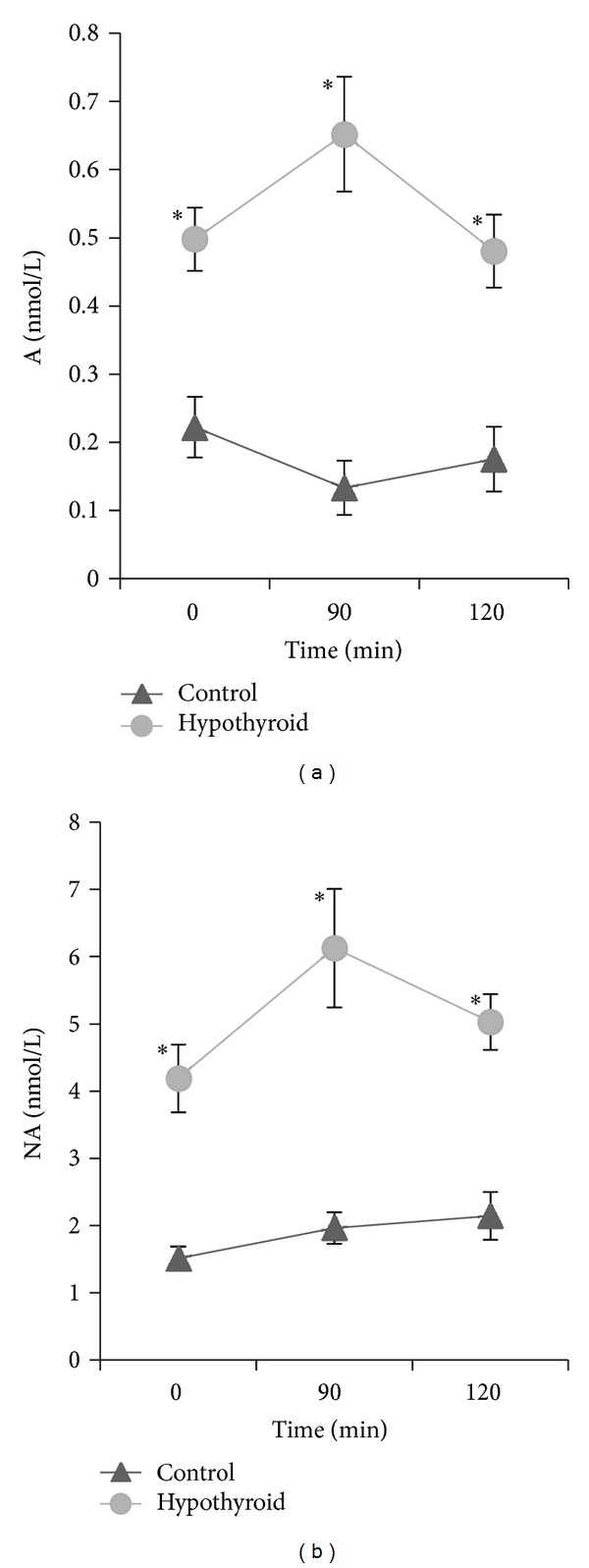
Mean values ± SE of fasting blood adrenaline and noradrenaline and their postglucose levels in the group of hypothyroid (hypothyroid) and healthy women (control). Asterisks denote differences between these groups; *P < 0.0002.

Both the initial concentrations of catecholamines in plasma and those measured after ingestion of glucose were significantly higher in women with hypothyroidism than in healthy women (Figure 3). Glucose injection caused statistically significant changes in the levels of plasma A and NA concentration in both groups. After 90 min of OGTT, there was a significant increase (*P* < 0.03) in the level of A in hypothyroid group, whereas in healthy group there was a decrease in the level of A (*P* < 0.03) compared to baseline values. In both groups, there were statistically significant increases in the level of NA after 90 min but they were considerably greater in hypothyroid group (*P* < 0.001) than in healthy group (*P* < 0.05). Moreover, the level of NA at the end of OGTT in both groups remained elevated compared to baseline at the border of significance in hypothyroid group (*P* = 0.055) and significantly higher in healthy group (*P* < 0.02). However, the level of A returned to baseline after 120 min in both groups. The maximal levels of A and NA in hypothyroid women were significantly higher than in control group (*P* < 0.0001 and *P* < 0.0001, resp.).

## 4. Discussion 

The principal finding of the study is that thermogenic response to the glucose ingestion in patients with hypothyroidism was much lower than in healthy subjects. This was demonstrated for the first time. The existing results concerning TEG in thyroid dysfunction are equivocal [7–9]. Both Acheson et al. [9] and Randin et al. [7] observed significant increases in RMR in hyperthyroid state compared with healthy subjects but did not report any significant changes in TEG. Randin et al. [7] showed that glucose-induced thermogenesis tended to be reduced after treatment of hyperthyroid patients when euthyroid state was achieved. Al-Adsani et al. [8] observed that slight changes in thyroid hormone levels affected the RMR but not the TEG. However, Ulas et al. [19] showed that energy expenditure during sleep was similar between hypothyroid and healthy subjects. Our studies have clearly showed that hypothyroidism associated with reduction in energy expenditure occurs not only in the case of RMR but also in the case of reducing TEG.

The study also demonstrates that thyroid hormone deficiency impairs glucose tolerance, which is in agreement with previous reports [10, 12, 20, 21]. Despite much larger amount of insulin released into the blood after glucose ingestion (IRIauc) in women with hypothyroidism, the glucose concentration in blood was high. The differences between the shape of the curves and the areas under the curves suggest postprandial insulin resistance related to hypothyroid state. Interestingly, none of the insulin-resistant indicators differed between groups. They revealed insulin-resistant state in both groups (potentially due to the relatively high BMI in both studied groups of subjects). However, the calculated indicators were unable to reflect the above-mentioned differences in the curves between groups.

Insulin appears to play an important role as the link between dietary intake and sympathetic nervous system (SNS) activity by its action in the brain ventromedial hypothalamus [22, 23]. It was also reported that not only obese insulin-resistant subjects display a blunted sympathetic neural response to glucose ingestion compared with insulin-sensitive individuals ([24] also [25, 26]). This was not confirmed by the present results but it should be noted that our study was conducted in subjects with more complex endocrine disorder.

In agreement with previous studies [27, 28], the basal plasma levels of adrenaline and noradrenaline were much higher in hypothyroid than in healthy women. Also the amount of noradrenaline released during OGTT was much greater in hypothyroid subjects than in healthy controls. This difference may result from stronger activation of sympathetic nervous system or/and decreased plasma noradrenaline clearance. Nedvidkova et al. [27] demonstrated that hypothyroid subjects have a weaker response to lipolytic stimulations of *β*-adrenoceptors. Increased sympathetic activity may be a compensatory mechanism to achieve an appropriate level of response tissues to stimulation. Thus, hypothyroidism is related to high activity of the sympathetic nervous system and to desensitization of adrenergic receptors [15–17, 29, 30].

In present study, the plasma level of adrenaline in healthy women decreased in response to glucose and/or insulin elevation as compared to baseline similarly as it was found in a previous studies [31, 32] whilst in hypothyroid group it showed an increase at the 90th minute of the test. Adrenaline is a hormone with a potent thermogenic effect. It is possible that weak thermal response to the intake of glucose in women with hypothyroidism causes increased secretion of adrenaline in order to enhance thermogenesis. Again, it may be compensatory mechanism by which adrenaline has to compensate the reduced postprandial metabolic rate. On the other hand, despite the fact that we studied the metabolism of carbohydrates, we cannot exclude the role of lipid metabolism in this metabolic play and its relationship with adrenergic activity.

Nevertheless, even significantly stronger adrenergic activation in women with hypothyroidism is not sufficient to compensate for the “catecholamine specific resistance” with diminished response of tissues to stimulation of beta receptors. That “catecholamine resistant” may be responsible for the reduction of the sympathetic nervous component of TEG. In addition, other factors such as glucagon, glucocorticoids, leptin [33], ghrelin, or obestatin [34] that could influence thermogenesis may also be modulated by thyroid hormone.

It can be concluded that in thyroid patients TEG is diminished and glucose tolerance is decreased while the adrenergic response to glucose administration is markedly greater. It can be speculated that these changes are related to decreased insulin sensitivity and responsiveness to catecholamine action.

## Figures and Tables

**Table 1:** Comparison of healthy and hypothyroid females: subjects' characteristics and results of OGTT test.

	Healthy subjects	Hypothyroid patients	*P* value
*n*	34	32	
Age (yr)	38.14 ± 1.85	38.53 ± 2.38	NS
BMI (kg/m^2^)	27.77 ± 0.86	28.99 ± 0.91	NS
TSH (mIU/L)	1.51 ± 0.22	5.03 ± 0.66	<0.001
fT3 (ng/L)	3.27 ± 0.11	1.25 ± 0.11	<0.0001
fT4 (ng/L)	12.43 ± 0.49	7.34 ± 0.57	<0.0001
Triglycerides (mg/dL)	52.16 ± 9.19	56.63 ± 5.27	NS
Cholesterol (total) (mg/dL)	150.33 ± 9.86	125.90 ± 8.14	NS
HDL-cholesterol (mg/dL)	51.84 ± 4.08	43.35 ± 3.89	NS
LDL-cholesterol (mg/dL)	92.51 ± 6.93	71.32 ± 8.23	NS
RMR (kJ/h/kg body wt)	3.17 ± 0.17	2.50 ± 0.07	<0.0001
TEG (kJ)	55.40 ± 7.32	19.68 ± 3.90	<0.0004
BG_auc_ (mmol/L·min)	188.41 ± 15.84	286.79 ± 23.65	<0.003
IRI_auc_ (mU/L·min)	4987.72 ± 583.88	7563.27 ± 863.65	<0.03
*A*_max⁡_ (nmol/L)	0.30 ± 0.07	0.69 ± 0.08	<0.0001
NA_max_ (nmol/L)	2.54 ± 0.30	6.42 ± 0.86	<0.0002

**Table 2:** Comparison of insulin-resistant indicators in healthy and hypothyroid females.

	Healthy subjects	Hypothyroid patients	*P* value
IRI_0_	9.34 ± 0.59	10.54 ± 1.04	NS
HOMA-IR	1.89 ± 0.13	2.22 ± 0.24	NS
QUICKI	0.35 ± 0.01	0.35 ± 0.01	NS
Matsuda index	5.28 ± 0.38	4.58 ± 0.45	NS
